# Collecting and Sharing Person-Centered AI Clinical Summaries Across Frailty Services Provided by the National Health Service and Voluntary, Community, and Social Enterprise: Protocol for a Co-Design and Feasibility Study

**DOI:** 10.2196/68511

**Published:** 2025-09-17

**Authors:** Kieran Green, Sheena Asthana, Oscar Josue Ponce-Ponte, John Downey, Joanne Watson

**Affiliations:** 1 Centre For Health Technology University of Plymouth Plymouth United Kingdom; 2 Mayo Clinic Rochester United States; 3 Torbay and South Devon NHS Foundation Trust Torquay United Kingdom

**Keywords:** artificial intelligence, AI, health care, future, medical documentation, data interoperability, person-centered care, multidisciplinary team, care coordination

## Abstract

**Background:**

Due to its association with multimorbidity, frailty gives rise to multidimensional needs for different services. Too often, patient preferences and service encounter information are not adequately shared.

**Objective:**

This developmental study aims to co-design, collect, and analyze encounter data from multiple community and primary-based multidisciplinary teams (MDTs) providing services for people with frailty to develop prototype large language models that can generate clinical and person-centered care summaries.

**Methods:**

Engaging stakeholders in 2 primary care networks, we will co-design the large language model to ensure it meets local needs and preferences as well as infrastructure, information governance, and regulation requirements. General practitioners will identify 50 patients with frailty requiring MDT engagement. Three consecutive encounters between the patients and different members of MDTs will then be audio-recorded. Recordings will be transcribed into text for concept design and model pretraining. These data combine stakeholder engagement insights to develop sensitive artificial intelligence (AI) models responding to stakeholders’ needs, workflows, and preferences. To generate the person-centered summaries, we will test 2 approaches to modeling the encounter data: graph-based modeling and hierarchical transformers. The AI-generated summaries will be compared to human-written summaries of the same encounter data and assessed for accuracy, quality, fluency, and person-centeredness. They will also be shared with the original MDT members for validation. We will capture inputs, processes, and outcomes across all key phases of the implementation journey to identify capability requirements, determinants of implementation (including key challenges and best practices to overcome them), and the value added by the technology.

**Results:**

This protocol aims to review implementation evidence and engage stakeholders in co-design. This work package will aid the development of contextually sensitive, longitudinal, and AI-generated person-centered summarization tools. Model development will aim to achieve longitudinal person-centered summaries tested against MDT standards. If deemed suitable for deployment, optimum ways of integrating these summaries into shared care records will be explored with local key system leaders. Model evaluations will provide conclusive insights into such technologies’ benefits and risks. As of August 2025, this study has not yet been funded, nor has ethical approval for the project been obtained. Consequently, dates of data collection and numbers of recruited participants are not applicable at this time.

**Conclusions:**

Our protocol provides a robust method of co-designing, evaluating, and implementing a longitudinal AI medical summary tool. Including key stakeholders at multiple stages facilitates an iterative development strategy that is designed to solve implementation challenges as they emerge. This project fits within our long-term vision to deliver a multimodal AI tool that saves clinicians time and deepens the health care professional–patient relationship. Future studies should include a larger patient sample, video-recorded health care professional–patient encounters, and a more extensive longitudinal evaluation.

**International Registered Report Identifier (IRRID):**

PRR1-10.2196/68511

## Introduction

### Background

Due to its association with multimorbidity, frailty gives rise to multidimensional needs for different services [1-4]. To reduce fragmentation and duplication of services and enhance person-centered care (PCC), information about patients’ clinical needs, service encounters, and preferences should be shared across multidisciplinary teams (MDTs). Too often, this information is neither adequately recorded nor shared [5,6].

Evaluation of a “what matters to you” (WMTY) initiative for patients with frailty in one of our local trusts found that WMTY conversations were often happening too late and in the wrong setting (hospital as opposed to primary or community setting), did not always involve the right people (eg, general practitioners [GPs] and voluntary sector and care workers), were subject to key trade-offs concerning staff time (eg, peripatetic community staff working in geographically dispersed settings entering encounters notes at the end of the day), and were not shared with other members of the MDT. Indeed, due to organizational pressures prioritizing patient flow, many WMTY conversations are overly focused on facilitating hospital discharge.

However, important conversations are happening every day with members of MDTs (GPs, community nurses, social workers, domiciliary carers, volunteers, and pharmacists) from the community to hospital settings. Too often, this information is neither adequately recorded nor shared, leaving an untapped source of valuable information about patients’ needs, beliefs, and personalized goals. Artificial intelligence (AI) tools such as large language models (LLMs) have been used to have a good effect on the summarization of synthetic patient-physician conversations [7]. However, these models tend to focus on clinical needs, drawing from notes written after a conversation, and offer little more than physicians' usual clinical summaries. Summarization models are needed to generate automatic summaries that include clinical needs and WMTY information from multiple MDT consultations without recall bias from health care professionals (HCPs).

Previous research and funding efforts have laid the groundwork for this proposal. We received support from the National Institute for Health and Care Research Invention for Innovation Funding At the Speed of Translation program to validate LLMs for generating automatic person-centered (PC) summaries of GP consultations [8]. Extensive engagement with patients and staff in 3 Plymouth practices and 1 Somerset GP practice informed our understanding that true PCC for this complex group of patients requires a better exchange of clinical and WMTY information into clinical records.

Our project proposes a feasibility study to co-design, collect, and analyze data to develop summarization models using an LLM to generate PC summaries of multiple primary and community service encounters among patients with frailty. Despite the good performance of current large language summarization models [7], implementation is challenging due to a lack of user engagement; ethical, legal, and data governance issues; and poor integration with existing data and workflows [9-11]. Importantly, intelligent data-driven care must also rely on unbalanced, missing, and incomplete clinical data drawn from imperfect real-world settings. Thus, careful preprocessing (eg, cleaning and substituting values) is essential to ensure good predictive modeling and prevent biases and unreliable outputs [12]. Our models, built on patients’ and key stakeholders’ preferences, values, and concerns, will be uniquely designed to generate interoperable PC summaries.

To this end, we will (1) engage stakeholders to understand technical, human factor, ethical, and regulatory barriers and enablers; (2) work with 2 primary care networks (PCNs) to audio record and transcribe primary and community encounters with 50 patients with frailty resulting in at least 150 audio-recorded conversations (eg, 3 encounters per patient); (3) develop and evaluate our LLMs ability to generate gold standard PC summaries that work for patients, carers, and MDT staff; (4) co-design implementation strategies and deploy pilot PC summary data into 2 PCNs; and (5) evaluate the summaries capacity to save HCPs’ time, improve PCC, and patient well-being.

### What Problem Are We Trying to Address?

The geography of older age in the United Kingdom is highly skewed toward coastal and rural areas, such as the Southwest Peninsula [13]. As people age, they experience more ill health and disability, resulting in higher rates of frailty and multimorbidity in these regions compared to urban areas. The British Geriatric Society, Age UK, and the Royal College of General Practitioners advocate for a proactive, PC, coordinated, and community-based response to frailty care [3]. However, individuals with frailty often navigate multiple care pathways, take numerous medications, and interact with various HCPs who do not communicate well with each other [5,6,14-17].

Key advocacy groups emphasize that improving communication between HCPs and emphasizing PCC is crucial to reducing service fragmentation. It is argued that PCC addresses patients’ emotional needs; aligns care with their spiritual and personal beliefs, social activity desires, and self-care goals [18-22]; and creates personalized goals that help reconcile conflicting health objectives across an MDT [23]. At a minimum, then, HCPs should ask, “What matters to you?” instead of “What is the matter with you?” However, in practice, there are concerns that WMTY conversations in acute hospital settings have become overly standardized due to organizational pressures prioritizing patient flow, reducing patient engagement time, and increasing compassion fatigue [14,24,25].

Moreover, these conversations often occur outside the hospital, between patients and their GPs, community nurses, social workers, domiciliary carers, pharmacists, volunteers, and informal carers. They are not adequately recorded or shared. This reduces care continuity across MDTs and overlooks valuable patient information that could enhance shared decision-making [26,27]. Realistically, the health system’s capacity for PCC is reduced because, in acute settings, the pressured context reduces implementation effectiveness, and in community or primary settings, HCPs lack the time to write summary notes covering WMTY and clinical needs.

Finally, many technical requirements remain, and there are potential challenges in building and implementing LLMs in health systems. Particularly, the ethical, practice, cultural, and information governance barriers to recording encounters require close engagement with patients, practitioners, and managers to ensure the feasibility and acceptability of data collection and that summarizations work for all end users [28-30]. Consequently, this project aims to understand and address these AI-related health care implementation gaps.

### Proposed Technological Solution

**Figure 1:**
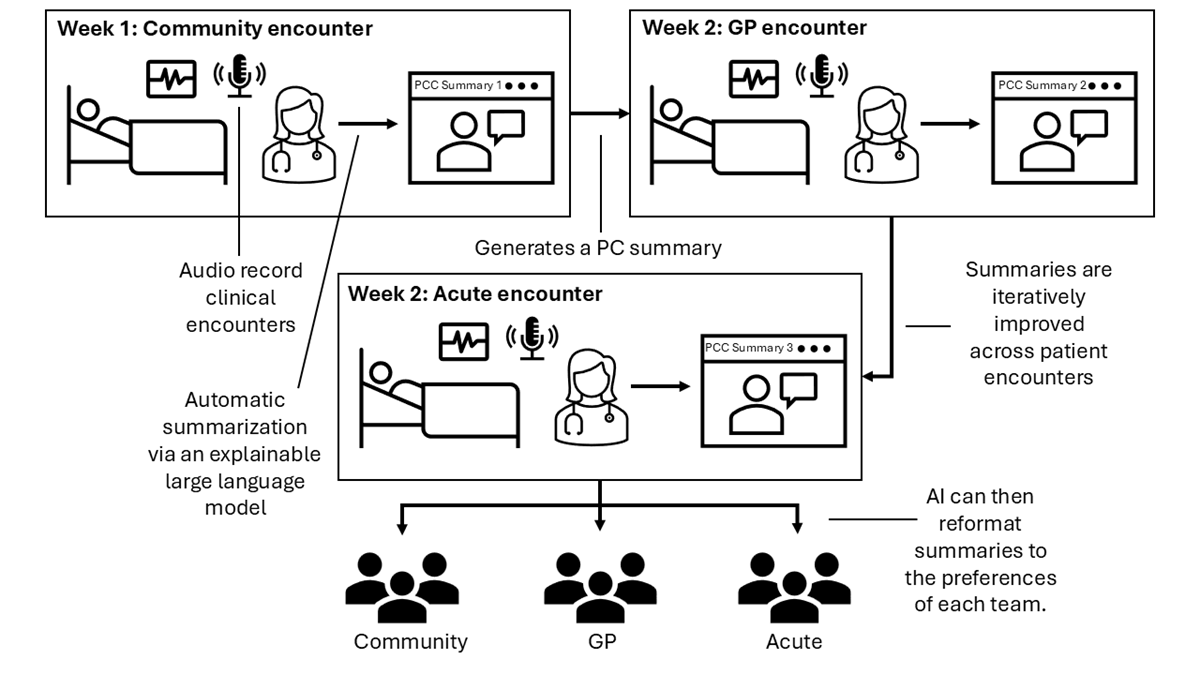
A diagram highlighting how the person-centered care (PCC) summarization tool would function. GP: general practitioner; PC: person-centered.

There is growing interest in generating medical summaries using LLMs to condense complex patient information from text or voice-recorded datasets [7]. For example, LLMs have also shown great potential in summarizing clinical reports [31]. However, only some have reached the quality required for clinical decision-making. In the United States, most summarization tools inform insurance and legal workflows and provide summary information for electronic patient records [32]. We similarly propose developing LLMs to summarize information rather than triggering clinical decisions. Even though multiple LLMs can generate high-quality clinical summaries [7], it is important to note that these models, such as the one used in this study [8], mimic what physicians do (ie, focus on clinical needs) as opposed to PC summaries. Our proposed solution addresses this gap in focus (Figure 1).

We evaluate whether LLMs can generate PC summaries from audio-recorded patient-physician conversations, fine-tuning the models to develop these summaries and then validating such models [8]. This modeling solves the problem of inadequate capture of PC information. However, achieving PCC requires developing or adapting LLMs for longitudinal summarization from multiple community or primary patient-physician MDT encounters. As part of an integrated, preventive, and community-focused care model, this ensures the meaningful transfer and acceptability of information across MDTs and hospital settings, and mitigates ethical, legal, and governance challenges

If successful, this technology will enhance PC community frailty care. Our PC and clinical data summaries will reduce administrative load, allowing more patient-clinician time in community or primary settings, which is crucial for PCC [5,33]. The synthesis and needs-based distribution of PC encounter data will also improve interoperability, enabling MDT practitioners to access relevant clinical and PC information instantly, reducing service fragmentation, and improving health outcomes [5].

Proactive, anticipatory care for older people with escalating levels of frailty identified in primary care, supported by sufficient capacity for holistic MDT assessment, personalized care planning, and tailored interventions, can also significantly reduce hospital admissions and facilitate earlier discharge [26,27,34-38]. In the National Health Service (NHS) Devon alone, the number of bed days occupied by patients over 75 years old for more than 21 days accounted for 238,340 bed days in 2022, equivalent to 244 beds. Our summarization models could play a significant role in supporting community-focused preventive care.

## Methods

### Overview

**Figure 2:**
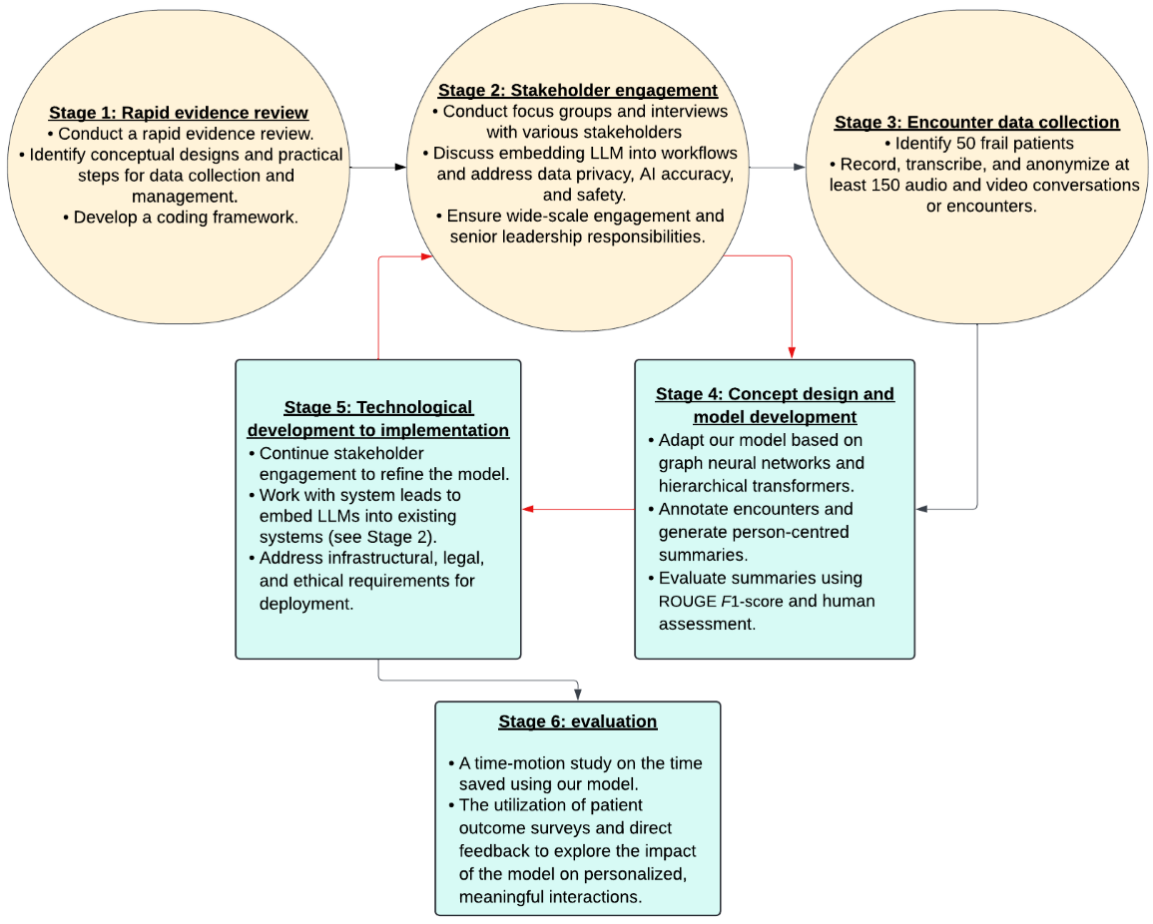
A flowchart summarizing the 5 research stages and their objectives. LLM: large language model.

We will develop model designs that respond to end users’ requirements regarding usability, interpretability, explainability, risk aversion, and privacy concerns. By integrating end-user feedback and understanding the sociotechnical context, we aim to ensure that our model supports clinical efficiency and wins the hearts and minds of patients and staff. This comprehensive approach will address the unique needs of local populations and foster the adoption of advanced health technologies in diverse health care settings (see Figure 2).

### Stage 1: Rapid Evidence Reviews

Embedding AI-generated PC summaries into care records is more than a technical exercise. There are feasibility concerns around the initial collection of data (eg, audio recording, staff and patient concerns around privacy, and possible impacts on the nature of encounters), its storage and related information governance requirements (eg, who owns the recording equipment and data), and how summary data should best be presented and integrated into the clinical records. We will undertake a rapid evidence review of AI models focused on patient-clinician research to summarize the current qualitative evidence of common ethical, practice, cultural, and information governance issues. These issues will center upon collecting encounter data (ie, patient-clinician conversation) and integrating summarization AI models into health care systems.

The review aims to understand the best practices and potential barriers to integrating automatic clinician-patient summaries from this rapid review of evidence. This understanding should help establish our models’ data collection, storage, and governance guidelines. It should also provide insights into potential low-fidelity model prototypes, preferences regarding the user-interface functionality, and the types of summaries that fit within existing end users’ workflows and implementation strategies. Finally, this review of evidence will provide a strong coding framework for analyzing the stakeholder engagement section in stage 2.

### Stage 2: Stakeholder Engagement

Two different focus groups will be held with a wide range of stakeholders to co-design and explore the ethical, practical, cultural, and information governance themes around the collection of patient-clinician conversations. Collected data from stage 2 helps form a key part of our participatory, co-design approach to model development and helps with later strategy development for integrating the functional prototype into a health care system. Findings from stage 1 inform the themes explored in the focus groups described subsequently.

Our first focus group’s key stakeholders (n=15) will include patients and carers, voluntary, community, and social enterprise **(**VCSE) representatives, GPs, community nurses, social workers, domiciliary carers, pharmacists, and acute health of older people teams. The discussions are likely to include the following topics: (1) how to embed the AI summarization model into health systems; (2) ethical issues around compliance with UK data privacy laws, AI accuracy and safety standards, measures to safeguard patient-clinician relationships, and clinical autonomy, and minimize biases and medicolegal liabilities; (3) cultural issues, including stakeholders past interactions with AI technologies and ensuring the technology is culturally sensitive and adapts to diverse needs; (4) practical discussions that aim to forecast and address barriers related to relevance, acceptability, and usability of AI summarization models; and (5) how they can be integrated into existing workflows, considering ease of use and integration into daily practices.

Insights from these discussions will guide the refinement of the AI summarization model and inform the development of implementation strategies. We aim to develop a strong understanding of ethical, practical, cultural, and information to code usable, functional LLM models that fit the participating health systems’ existing workflows, cultures, and concerns. From this discussion, we may realize it is important to code specific added measures to safeguard against harm to patient-clinician relationships, clinical autonomy, recall biases, and other medicolegal liabilities.

The second focus group (n=15) will consist of local information governance specialists, IT services, data specialists, managers (hospital, primary care network, and care homes), local authorities, integrated care boards, and regional NHS regulatory boards. This group aims to produce agreements, understandings, and adaptations to improve our models’ viability and likelihood of implementation. The questions will likely explore ensuring wide-scale engagement, senior leadership responsibilities, adapting the basic concept model (Figure 2) to meet localized infrastructural needs, who owns the data collected, where it should be stored, and the potential of these data to be shared and accumulated across multiple nodal points in the MDT. These group discussions then shape model development to meet local data governance standards and agreements on who across the MDT will own the data produced by our LLM, where the data will be stored, and how best to ensure its interoperability. It should also identify senior leadership oversight and responsibilities, and required inputs from MDT managers through development and later implementation.

We know that technologies can fail because of poor design, which stems from a lack of understanding of the actual requirements of the end users. Thus, this phase involves embedding co-design into the project and winning hearts and minds. The stakeholder engagement stage aims to enhance our co-design efforts, creating a truly PC model that addresses the needs of patients, staff, managers, and infrastructure specialists. By improving our co-design process, we can foster support and understanding, ultimately aiding in the successful development and implementation of our model.

### Stage 3: Encounter Data Collection

Audio recordings of encounters between different members of an MDT team and patients with frailty will occur in 2 different PCNs, one between months 6 and 12 and another between months 9 and 15. The first PCN is more experienced in cross-team data sharing and deploying AI solutions and is more prepared to test the feasibility of audio recording encounters than the second PCN, which is more digitally immature. If necessary, access to these patient participants may also be supported by the Clinical Research Network Southwest Peninsula, which can assist the team in contacting other GPs who can help identify suitable and eligible research participants.

Working with GPs, we will identify 50 patients with frailty who require MDT engagement and ensure that MDT members know the project, its aims, methods, and data management protocols. To mitigate the limitations posed by our relatively small sample size, we will apply purposive stratification, ensuring a balanced representative sample across key frailty-associated factors, such as differing clinical frailty scores, typical comorbidity clusters, and social determinants.

To capture a patient’s data from multiple encounters between them and their community-based HCP, the research team will liaise closely with each patient participant and their community carers, encouraging them to provide updates on each future engagement over 2 months to record 3 to 5 consecutive encounters. Each patient will need to agree to participate in at least 3 encounters.

When the researcher is informed of an upcoming encounter, a consenting HCP or patient will notify the researcher of the time, date, and location. The researcher will then attend and record the conversation using high-quality audio-recording devices (Shure SM7B microphones). Patients will need an individual identifier to link encounter data from different MDT members and validate summarizations. Along with these identifiers, each encounter will be identified sequentially, from the first recording to the last. Each patient will participate in at least 3 encounters with HCPs (MDT members), resulting in approximately 150 conversations or encounters. All recordings (with anonymized identifiers) will be transcribed into text for concept design and model pretraining.

### Stage 4: Concept Design, Model Design, and Validation

Data from stages 1 to 3 will be collated, synthesized, and shared with the model developers to adapt an AI summarization model that responds to different stakeholders’ needs, workflows, and preferences.

PC AI summarization has been previously defined by Ponce Ponte [8] as a model capable of summarizing clinical issues as well as patients’ preferences and values. The exact themes of patients’ preferences and values will be derived from further public and patient involvement and engagement (PPIE) with patients and staff in 3 GP practices involved in prestudy PPIE, across 2 counties in southwest England.

Annotators will be trained to generate PC summaries based on audio-recorded conversations between patients and HCPs. This will be carried out following the sequence of a patient’s encounters with their HCP’s (start visit 1, continue to visit 2, etc).

Two approaches have successfully generated automatic summaries from multiple documents: (1) graph-based modeling and (2) hierarchical transformers. On the basis of these approaches, we will adapt our patient-centered AI summarization model [8]. Multiple document summarization approaches are necessary because conversations between patients with frailty and MDT members will accumulate over time. The first model will be developed using graph neural networks, which follow an extractive summarization approach. We will develop a graph structure by encoding words and sentences and generating words and sentence nodes related to an edge. The edge represents how strong their relationship is and will be calculated with the term frequency and inverse document frequency method [39]. This learning will be done per encounter via an iterative process. Once a second encounter is completed, the process will be conducted again. This time, a third level of node will be created, the document node, and this will be included in the learning process to generate a new graph structure. This will continue until the last encounter. After every encounter, we will use the graph structure to extract sentence nodes, follow a trigram blocking for decoding, and generate an extractive PC summary. The training will be done to predict the sequence of sentences from the gold-standard PC summary.

The second model to be tested is based on a hierarchical model. We will develop this by comparing and integrating multiple pretrained LLMs, for example, LLaMA (version 3.1), Falcon, Mistral, and bidirectional encoder representations from transformers and its variants (BioBERT, etc) [40]. Our method to compare these models will require evaluating each using a Recall-Oriented Understudy for Gisting Evaluation ROUGE *F*_1_-score, assessing their capacity to compare PC themes. We will then add a hierarchical adapter on the encoder layer to contextualize sentence semantics or meaning. The overall objective of the model is to predict the next word. Then, to generate the PC summary, we will use retrieval-based selection to choose the most salient content using sentence similarity.

We will automatically evaluate the quality of these summaries using ROUGE *F*_1_-score. This metric compares the AI-generated summaries against our gold-standard summaries. Trained, blinded evaluators will perform human-based evaluations. They will assess their informativeness, fluency, quality, and person-centeredness (number of PC themes captured by the summaries).

### Stage 5: From Technological Development to Pilot Implementation

After stage 4, we continue to engage with key stakeholders until we reach a saturation level whereby the model’s development either deeply reflects the complexity of localized needs and preferences in the concept design, making the technology more suitable for deployment and integration, or is deemed unfeasible.

Doing this will require repeated prototype engagement and daily discussion sessions with HCPs using the system. This session will be conducted specifically with our first focus group, including patients and carers, VCSE representatives, GPs, community nurses, social workers, domiciliary carers, pharmacists, and acute health of older people teams. Each session will include an introduction explaining the purpose of improving the tool and meeting clinical needs, a brief demonstration, an opportunity to test and use the prototype in small groups, and then report on their experiences. The group of 15 participants will then be subdivided into 5 groups of 3 (20%) participants each and asked to complete the following tasks: use the device to generate a summary based on a prerecorded pseudoanonymized patient-clinician interaction, review its associated AI-generated summary, edit it, and integrate it into an electronic patient record. After completing these tasks, prototype engagement participants will return to the larger focus group to share their experiences, challenges, and suggestions. Topics will likely include ease of use and intuitiveness, clarity and relevance of the summaries, to what extent they could imagine the technology fitting within existing workflows, responsiveness of the tool to correction and feedback, consistency and acceptability of the terminology, and recommendations for enhancements. This process will be repeated at least twice but primarily until saturation is reached, which is defined as broad HCP satisfaction with the usability of the devices.

After saturation, if stakeholder support for these model implementations exists, the research team will then work closely with the second focus group from stage 2, which includes local information governance specialists, IT services, data specialists, managers (hospital, primary care network, and care homes), local authorities, integrated care boards, and regional NHS regulatory boards (n=15). Across 3 sessions, this group will aim to create bespoke implementation strategies to reflect the 2 PCN systems’ infrastructure limitations, support teams’ specific needs embedding the models into daily practice, and deliberate methods of providing an ongoing support system for later pilot-testing and wider-scale deployment. In session 1, the group outlines a strategy to implement our system into their existing workflows and IT systems and identifies necessary adjustments to enable the model’s integration. Session 2 will ensure that the previous strategy meets regulatory and ethical guidelines, and finally, session 3 outlines a package of technical support and seeks to achieve agreement on responsibilities and channels for accessing this support channel and who provides the training.

After ensuring that both adopters and key stakeholder implementers are happy with the technology and that it could be implemented into their health systems, we will reach out to each clinical team engaged in its implementation, alongside local PCN technology deployment and support specialists, to finalize an agreed upon implementation and evaluation timeline and ensure they are happy with our proposed evaluation objectives and metrics (see stage 6 described subsequently). Once approved, we will engage each team and deliver essential training on using the system. This training will include preparing the microphone, recording, creating the PCC summary, sharing the summary in the electronic patient record, and building on past summaries with new clinical encounters for the same patient. Once all teams are happy with setting up, creating, sharing summaries, and accessing support, final verification of resources, allocation training, and support will be conducted, and then the AI system will go live.

### Stage 6: Evaluation of Implementation

#### Overview

The final part of this study will evaluate the implementation of the AI summarization model within a health care setting. This evaluation will explore whether the summarizations positively impact HCPs and patients. To do this, we will analyze the impact on clinical workflows, patient outcomes, and perceptions of the impact on perceived workload; assess the usability and acceptability; and determine whether any biases or medicolegal risks emerge and if the system positively impacts personalized and meaningful patient-clinician interactions and relationships.

#### Time-Motion

This evaluation will first address the impact on clinician workflows. The process will include a time-motion study with 9 HCPs based across primary care clinic (n=3, 33%), intermediate care (n=3, 33%), and domiciliary care (n=3, 33%). We will observe HCPs in their respective work settings under 2 conditions for each participant. First, we observe HCP-patient interaction and their documentation process with standard non-AI methods. Second, we observe HCP using the AI PCC summarization system.

To do this, the research team breaks down the process into stages, one measuring normal practice workflow. In normal practice, stages include (1) reviewing previous records before engagement; (2) engaging in HCP-patient interaction; (3) documenting the consultation in the electronic patient record postencounter; and (4) HCP submitting, sending, or embedding the summary into the electronic patient record. Our AI system includes the following stages: (1) reviewing AI PCC summary before a patient engagement; (2) setting up the recording device; (3) engaging in the HCP-patient interaction; (4) packing away the device; (5) HCPs checking the summary for acceptability; and (6) HCP submitting, sending, or embedding the summary into the electronic patient record. We will then analyze the data to identify (1) whether the implementation of our system reduces the time taken and (2) if there are further opportunities to streamline and better embed the technology into a care pathway [41,42].

#### Acceptability and Usability

To evaluate the model’s usability and acceptability, we also apply a longitudinal mixed method approach involving semistructured interviews (n=15) and the System Usability Scale (SUS; n=20). Participants include HCPs (eg, general practitioners, community nurses, social workers, and domiciliary carers). Longitudinal interview and questionnaire assessments will occur at 3 time points: baseline, 1 month after intervention, and 6 months after intervention.

Semistructured interviews will be conducted face-to-face or via videoconferencing platforms (n=15) to receive feedback from HCPs to help understand their perceptions of whether the model has streamlined the documentation, perceived workload, and ease of use; how effectively the model adheres to ethical, privacy, and information governance standards; and any challenges encountered and suggestions for improvement. Importantly, as interpretability and explainability are now a mandatory regulatory requirement in various regulatory contexts, such as the European Union, participants will discuss their comprehension of the rationale behind AI-generated recommendations, clarity and interpretability of outputs, and ability to identify potential inaccuracies or biases. This identification of challenges will be documented, and the model adapted accordingly [43].

Immediately after the interview, participants will complete the SUS, a validated 10-item questionnaire that measures perceived usability. Responses are scored between 0 to 100. Higher scores indicate better usability [44-46]. Finally, this process will be repeated in our longitudinal assessments at 3 time points: baseline, 1 month after intervention, and 6 months after intervention. Together, these provide a helpful insight into the model’s usefulness as it becomes more deeply embedded into day-to-day practices.

#### Impact on PCC

To evaluate the impact of AI summarization on PCC, interviews with HCPs (n=10) will assess their perceptions of how the system influences their ability to provide PCC. These interviews will explore, when compared to a standard practice, if HCPs perceive an improvement in their capacity to understand patient priorities through the summaries, whether the summaries enhance their engagements and enable what they regard as more PCC outcomes, helped patients become active participants, and whether they felt they had the necessary skills to use the WMTY summaries [47].

#### Patient Benefit Measures

To evaluate the impact of the summarization tools on the quality and delivery of PCC, we will use multiple self-reported and validated measures, each capturing different aspects of potential patient benefit. Numerous tools are available for these purposes; however, the EQ-5D, collaBORATE tool, and Meaning in Life Questionnaire have been chosen as they are fast, reliable, and sensitive to changes over time. Two groups are needed to evaluate the impact: a control group (standard care without AI summaries) and an intervention group (care incorporating the AI-generated PCC summaries). Patients in both groups will be matched based on demographic and clinical characteristics to minimize confounding variables. Longitudinal assessments will occur at 3 time points: baseline, 1 month after intervention, and 6 months after intervention.

The EQ-5D is a standardized health-related quality-of-life measure that evaluates mobility, self-care, usual activities, pain or discomfort, and anxiety and depression [48]. The collaBORATE measure of patient-reported shared decision-making will help us evaluate how the AI-generated PC summaries facilitate the development of care plans aligned with patient values and preferences [49]. Finally, recognizing PCC involves developing personalized goals that produce health outcomes and improving a patient’s perception of meaning in life [21]. The Meaning in Life Questionnaire assesses the extent to which the AI system influences patients’ sense of meaning [50,51]. Patients in both groups will complete this questionnaire at baseline, 1 month, and 6 months postintervention.

A total of 100 participants will be recruited—50 (50%) participants in the control and 50 (50%) participants in the intervention group. Participants will be sourced from the 2 PCNs as in other stages of the study.

#### Risks

Participants’ well-being will be prioritized with minimal risks through careful study design and continuous monitoring. Participants can contact the principal investigator or data protection team with any data collection concerns. Interviews are scheduled to last 40 to 60 minutes, with focus groups lasting between 60 and 120 minutes. Recognizing that these time lengths may be demanding for older people and busy frontline clinicians, we implement the following mitigation strategies: provide flexible scheduling to minimize disruption; clear objectives and structured questions to make discussion efficient; offer frequent breaks to reduce fatigue, allowing people to step away if necessary; and provide options for remote participation. The facilitator will also continuously monitor participants during the session and be prepared to pause or end the session if fatigue is noticed. All interviews and focus groups will be conducted in private rooms to ensure privacy.

This research focuses on co-designing our AI models for end users, ensuring functionality and acceptability of audio recording clinical encounters to patients, clinicians, and health systems, and ensuring that these devices ethically and safely share data across an MDT. Consequently, the interviews avoid areas of cultural and psychological sensitivity and are low risk. We also expect that MDT members and hospital managers will have full ability to understand and consent, given their professional involvement in the program, its implementation, and associated improvements.

However, the participant pool will involve an older adult population, often with multimorbidity conditions, including dementia, learning disability, or mental health issues. Therefore, additional protection will ensure their safety and comfort, such as providing breaks and a supportive environment. In distress, participants will be directed to appropriate support services such as the Samaritans. The researcher will also follow a careful protocol to assess capacity to make decisions in line with the Mental Capacity Act 2005, which states that “a person must be assumed to have capacity unless it is established that he or she lacks capacity.”

Patients and staff may also disclose risks to themselves or others, such as professional malpractice and complaint issues. Thus, the research team will be updated with their mandatory safeguarding training. They will also have completed a Disclosure and Barring Service check before working with study participants. Using honorary NHS contracts, the research will also use NHS email accounts to ensure confidentiality and security when conveying sensitive material or collected data. They will also have a policy in place should the researchers become aware of any risks, safety issues, or concerns (ie, self-injury or suicidality) during interviews or focus groups. This escalation policy is taken from Devon’s Safeguarding Adults Partnership.

### Study Governance

A research ethics committee will review the study, and the team will obtain approval from the Health Research Authority through the Integrated Research Application System to protect participants’ rights, safety, dignity, and well-being.

A research management group will oversee the research. The research management group will be a learning partnership between 2 NHS integrated care boards, their subsidiary PCNs, and the university. This partnership group will oversee the delivery and direction of the research and meet regularly (bimonthly, every 2 weeks). Its membership will include a GP from each PCN, a deputy director of innovation and transformation, a chief clinical information officer, and senior researchers at the university’s center for health technology. Distributing the leadership ensures commitment and buy-in from the 2 organizations and a true partnership initiative.

### Ethical Considerations

This project has not yet been submitted to an institutional review board or the NHS Research Ethics Committee.

#### Informed Consent

HCPs and other associated stakeholders (n=30), excluding patients and carers, will be approached initially via an appropriate email introduction for an interview or focus group. These stakeholders will be identified within the 2 PCNs previously discussed. Initial contact will likely be bridged via a collaborating GP or one of their administrative team members. The introductory email will clarify the study’s aims and objectives, the organization’s consent to participate, the voluntary nature of participation, and the date by which they can withdraw their contributions without providing reasons or facing any consequences.

A participating HCP will first approach older patients (n=50) from the 2 PCNs. Using the participant information leaflet, they will provide an overview of the research. If interest is shown, a team researcher will provide further details, including the purpose of the research, a participant information sheet, and a consent form. These sheets will be adapted to ensure readability for older people, breaking down the stages of co-design, recording encounters, and evaluation. The researcher will explain the participant information sheet, the importance of the participant’s input, data use, sources for further information or queries, and withdrawal options. After 24 hours, participants can ask additional questions and provide consent.

#### Data Management

The research team will comply with the Data Protection Act 2018 requirements regarding collecting, storing, processing, and disclosing personal information. Electronic personal data (eg, consent forms) will only be stored on the university’s mainframe computer network, which is secure and password-protected. Paper copies of personal data will be destroyed using the university’s confidential waste management service. Each participant will be given a unique ID number that will be kept separate at the first stage of data storage. The key to IDs will be held in a safe and password-protected file on the university’s secure main computer drive. This password will not be shared with anyone except the principal investigator and their research assistant. The local custodian of the data will be the principal investigator at the university. In line with current recommendations around NHS-based research, all personal data are destroyed 3 months after the study’s completion.

Audio-recorded interviews and clinical encounters will be stored securely on PCN services and computers. The research team will transcribe all audio recordings, and all data will be pseudonymized. All audio-recorded conversations will be anonymized using Adobe Audition audio-editing software to silence location names and alter the voice’s pitch. Once anonymized encounters are transferred securely to the university’s OneDrive database on password-protected, encrypted university laptops. Subsequently, anonymized clinical encounters will be shared with the research team (n=4) and inputted into the AI model (n=150). No confidential or patient-identifiable data will leave an NHS setting at any stage.

Similarly, data related to developing and training LLMs will be stored securely on the university’s secure encrypted servers, including model configurations, training data, and evaluation metrics. Safeguards will be in place to ensure the integrity and confidentiality of the modeling data following institutional data security policies. In line with current recommendations around NHS-based research, this research data will be retained for 5 years after the study. After this, it will be archived in the university’s electronic archive and research repository and another suitable national or international data repository for long-term curation.

## Results

### Stages 1 and 2: Review and Stakeholder Engagement

The rapid evidence review will provide detailed qualitative insights regarding feasibility, privacy, interpretability, explainability, and information governance concerns. This understanding establishes foundational guidelines for data collection, storage, model development, and governance strategies.

Our stakeholder engagement groups then aim to produce contextually sensitive insights that enable the development of our model and implementation strategy that reflects the needs, capacities, and constraints. Our first discussion aims to develop a strong understanding of ethical, practical, cultural, and information to code usable, functional LLM models that fit the participating health systems’ existing workflows, cultures, and concerns. For example, from our first stage 2 discussions, we may realize it is important to code specific added measures to safeguard against harm to patient-clinician relationships, clinical autonomy, recall biases, and other medicolegal liabilities. Our second discussion, with managerial and technical stakeholders, aims to shape model development to meet local data governance standards across the MDT, establishing clear agreements regarding data ownership, storage, and interoperability.

### Stages 3 and 4: Model Development and Validation

This stage collects audio recordings from approximately 150 MDT encounters with 50 purposively sampled patients with frailty. By collecting 3 consecutive encounters with each patient, we will yield a robust dataset for beginning to test, train, and validate our summarization model. This validation process explores the models capacity to improve the HCPs’ understanding of their patients’ PCC needs over time. Our dataset will also represent a range of frailty levels, comorbidity clusters, and demographic characteristics, ensuring comprehensive model training. This representativeness should suffice for early-stage model training.

Graph-based and hierarchical transfer models will be developed and compared after encounter collection. Specifically, we will produce a comparative performance metric, using ROUGE 1, for each model against a human-generated gold-standard summary. The results will indicate the strengths and weaknesses of each approach, guiding further model refinement. Human evaluators will also qualitatively assess the AI-generated summaries, providing insights on their accuracy, quality, fluency, and person-centeredness. Overall, this process will identify which model most effectively summarizes PC information from longitudinal MDT encounters and carefully reflects the unique needs of PCNs and their related community assets.

Through repeated engagement sessions, prototyping evaluations by MDT members will refine the usability and capacity of implementing the AI summarization tool. This iterative prototyping, codeveloped with local stakeholders, should enable sufficient alignment with workflow integration, IT system compatibility, regulatory compliance, and necessary training and support infrastructure for implementation. This process will also help identify necessary training needs. Stage 5 should produce an implementation strategy and training program that reflects consultation with HCPs and management teams. If a feasible prototype and implementation strategy are not achieved, a comprehensive report will be produced outlining key problems and potential solutions for implementation attempts.

Finally, if stage 5 is successful, our stage 6 evaluation will clarify the time saved, usability, acceptability, and the patient and clinical benefits of the model. Time-motions will evidence the potential time saved compared to HCP’s usual documentation processes. The SUS scores and thematically analyzed qualitative data identify the model’s degree of usability and acceptability. In addition, analyzed interview data indicates whether HCPs perceive an improvement in their PCC practices. Finally, we will understand the benefit of the tool for patients, such as its impact on quality of life, meaning in life, and the patient’s perception of PCC, via multidimensional surveys.

## Discussion

### Anticipated Findings

Using audio equipment to record clinical encounters and automatically summarize them to elicit key PC information can reduce the administrative burden on staff across the MDT [7,52,53]. Technology development in this area should allow staff to spend more time on face-to-face engagements and less on administrative tasks. It may afford practitioners more time for PCC-related training schemes. Furthermore, with less need to record the notes and thus more time to allow conversations to flow naturally, it may act against tendencies for PCC to become overprotocolized regarding questions asked and notes taken [14,33,54]. In essence, emerging summary models can increase the time HCPs engage in meaningful patient interactions, thus improving patient reports of PCC [14].

However, this project aims to make an exciting development in LLM modeling to support PC, community-focused health care for older adults. At present, summary notes are being developed to combine diagnostic decision support systems to highlight the most relevant clinical information [55], developing intelligent methods to structure, organize, and improve the accuracy of summaries [56] and reduce critical fact omissions and hallucinations [57]. We have begun exploring how to enhance the relevance and usability of summaries for different clinical teams [58,59]. However, these models mimic current summarization practices and do not generate PC summaries. Alternatively, our model integrates longitudinal encounter summaries to iteratively improve the PC summary and adapt the summaries for varied MDT needs.

We aim to produce effective models to support integrated PC frailty care pathways. These summaries would provide HCPs with an iteratively improved understanding of “what matters” to a patient, developed through successive encounters. HCPs, no longer reliant on poorly transmitted or incomplete WMTY data from across an MDT, will receive an instant, up-to-date, meaningful PC summary in a format that reflects HCP and patient preferences. This should make it easier to organize and prioritize the MDT’s objectives based on the superordinate preferences and values of the patients [5,23]. This enhancement in care coordination is expected to improve clinical outcomes, enhance patient experiences, reduce service fragmentation, and increase the meaningfulness of the work for health care staff [22,60,61].

In addition, this project helps improve data-sharing capacities across the MDTs (voluntary sector and primary, acute, and social care) and the implementation of novel AI and audio-recording technologies [62]. This integration is invaluable with a growing array of data points from wearables, smart devices, and environmental sensors that could help identify and support preventive health care (eg, intervening at the early signs of deterioration and spotting anomalies in usual behaviors, such as diet) [63].

### Limitations

Potential limitations to this study can be identified. Most important is the limited sample size of 100 patients (control group: n=50, 50%; intervention group: n=50, 50%). Such a sample size may limit the generalizability of our findings, especially given the heterogeneous and complex nature of a person with frailty’s health presentations when they encounter the health system. In a bid to mitigate this potential generalizability, such as UK studies on population with frailty, we use purposive stratification for its small sample, ensuring our research reflects various key characteristics, such as severity, age, comorbidity clusters, and living circumstances [64-66].

In addition, conducting our stakeholder engagement and co-design with only 2 PCNs while making the project more feasible and inexpensive may limit the generalizability of our results for similar development and implementation projects in different health systems or elsewhere in the United Kingdom. Nonetheless, the study design includes a diverse patient population, various clinical settings, and a range of key stakeholders, from macropolicy to meso-organizational and microclinical levels.

Moreover, the success of this project depends on overcoming significant technical challenges. For example, aiming to generate increased PCC summary complexity across iterative engagements will require continuous feedback and human assessment to ensure accuracy and reliability. In addition, the United Kingdom’s NHS has fragmented electronic patient record systems, which often differ from primary care, social care, voluntary sector, and systems records. This variation in functionality and compatibility will present a challenge for effectively transmitting these data across different platforms, particularly at the implementation stage. Our co-design and stakeholder engagement section seeks to overcome these challenges using interim solutions, such as a stand-alone application or partial integration.

Our study lacks any interim results, except for some nonresearch stakeholder engagement that indicated the system functionality preferences of GPs and patients. Consequently, the methods presented in this protocol paper may require adaptation to ensure they apply to real-world settings. To mitigate these concerns, the study prioritizes building strong relationships with selected PCNs, encouraging active participation in study design and model co-design.

### Future Directions

Recognizing some of the key limitations of this study leads us to fruitful pathways for developing this research. First, future studies should include a larger and more diverse patient cohort. Recruiting multiple PCNs in varied urban, rural, and socioeconomic settings and doubling the participant pool should aid generalizability in future phases. Second, a future longitudinal study spanning 6 months to a year could also explore the longer-term impact of these summaries. Additional qualitative interviews could also explore how these summaries adapt MDT workflows and clinical management across time. Finally, during a future validation of the PC summaries, integrating a wide range of stakeholders, including patients and caregivers, could strengthen the person-centeredness of the models’ outputs.

We envision a future AI-enabled secure platform whereby each clinical interaction is seamlessly video and audio recorded and uploaded. On this platform, generated from previous encounter data and existing data in patient records, up-to-date PC summaries will be easily accessed by HCPs before each consultation in prefigured and personalizable formats. Patients will be notified of the summary provided to the HCP, so they know the information they need to share and what they do not. This should prevent patients from having to repeat their stories.

The AI-enabled platform, accessible by patients and authorized staff, family, and friends, will also have a ChatGPT or other online LLM-styled chatbot so they can ask questions about the patient record and request the AI to create multimodal summaries based on their moment-to-moment needs. For example, one may request to receive longer and more detailed PCC summaries, TikTok-style video summaries of past clinical encounters, a medication plan, or soundbites that can be played through a person’s phone or smart speaker. On the basis of our previous PPIE, this platform could also automatically generate meaningful graphs highlighting the interlinkages between psychosocial concerns and medical ailments. However, achieving this will require several future projects.

The AI tool could help ensure that care plans aim for medical goals, meaning in life, and connectedness to others when illness drastically affects their mental capacity. For example, with a long-term dataset spanning a lifetime, an AI could prompt HCPs based on previous interactions and suggest personalized care methods. The model would work to understand *the spirit* of a person—an essence of who they are and what they care about, across time. Of course, these needs and preferences would need to be evaluated with the understanding and support of the caregivers and careful and close attention to the patient’s response to the types of support they receive. However, this approach may again prove fruitful.

Developing the AI capacity and digital readiness to implement these technologies successfully will also necessitate radical and positive changes to how information is owned, distributed, and integrated across the NHS and other health care service providers. For example, we envision our model embedded within a broader and regional orchestration layer for the input and sharing of multiple remote monitoring devices and the capacity to receive and effectively share information between the voluntary sector service to create opportunities for a greater depth of AI summarization. This means that our summarization tools will also be able to consider and summarize information from other AI systems and databases that monitor such things as vital signs, gait, and activities of daily living. Importantly, these data would be owned by the patient.

### Conclusions

Incorporating AI-generated PCC summaries from audio-recorded encounters could reduce the time clinicians spend on notetaking, improve the quality of notes, and allow these notes to meet the various needs of different members across the MDT. We are developing a unique approach to LLM development by facilitating highly interoperable and time-efficient PC notes. The study can also elicit key understandings of implementing AI technologies in digitally immature, demographically aging coastal regions. Finally, there is great scope for the development of these technologies to become increasingly personalized to the needs of different staff in a variety of formats, and with the AI model expanding to receive inputs from various sources, such as nursing homes, personal writings, telephone calls, and existing PC information documents.

## Abbreviations

AI: artificial intelligence
GP: general practitioner
HCP: health care professional
LLM: large language model
MDT: multidisciplinary team
NHS: National Health Service
PC: person-centered
PCC: person-centered care
PCN: primary care network
PPIE: patient involvement and engagement
ROUGE: Recall-Oriented Understudy for Gisting Evaluation
SUS: System Usability Scale
VCSE: voluntary, community, and social enterprise
WMTY: what matters to you

## References

[1] Mitnitski AB, Mogilner AJ, Rockwood K (2001). Accumulation of deficits as a proxy measure of aging. ScientificWorldJournal.

[2] Fried LP, Tangen CM, Walston J, Newman AB, Hirsch C, Gottdiener J, Seeman T, Tracy R, Kop WJ, Burke G, McBurnie MA (2001). Frailty in older adults: evidence for a phenotype. J Gerontol A Biol Sci Med Sci.

[3] Turner G, Clegg A, British Geriatrics Society, Age UK, Royal College of General Practioners (2014). Best practice guidelines for the management of frailty: a British Geriatrics Society, Age UK and Royal College of General Practitioners report. Age Ageing.

[4] Majnarić LT, Babič F, O'Sullivan S, Holzinger A (2021). AI and Big Data in healthcare: towards a more comprehensive research framework for multimorbidity. J Clin Med.

[5] Berntsen G, Høyem A, Lettrem I, Ruland C, Rumpsfeld M, Gammon D (2018). A person-centered integrated care quality framework, based on a qualitative study of patients' evaluation of care in light of chronic care ideals. BMC Health Serv Res.

[6] Olsen CF, Debesay J, Bergland A, Bye A, Langaas AG (2020). What matters when asking, "what matters to you?" - perceptions and experiences of health care providers on involving older people in transitional care. BMC Health Serv Res.

[7] Van Veen D, Van Uden C, Blankemeier L, Delbrouck JB, Aali A, Bluethgen C, Pareek A, Polacin M, Reis EP, Seehofnerová A, Rohatgi N, Hosamani P, Collins W, Ahuja N, Langlotz CP, Hom J, Gatidis S, Pauly J, Chaudhari AS (2024). Adapted large language models can outperform medical experts in clinical text summarization. Nat Med.

[8] Ponce Ponte O (2024). The validation of an artificial intelligence model to generate automatic patient-centred summaries of GP consultations. University of Plymouth.

[9] Murdoch B (2021). Privacy and artificial intelligence: challenges for protecting health information in a new era. BMC Med Ethics.

[10] Chomutare T, Tejedor M, Svenning TO, Marco-Ruiz L, Tayefi M, Lind K, Godtliebsen F, Moen A, Ismail L, Makhlysheva A, Ngo PD (2022). Artificial intelligence implementation in healthcare: a theory-based scoping review of barriers and facilitators. Int J Environ Res Public Health.

[11] Wang L, Zhang Z, Wang D, Cao W, Zhou X, Zhang P, Liu J, Fan X, Tian F (2023). Human-centered design and evaluation of AI-empowered clinical decision support systems: a systematic review. Front Comput Sci.

[12] Hassler AP, Menasalvas E, García-García FJ, Rodríguez-Mañas L, Holzinger A (2019). Importance of medical data preprocessing in predictive modeling and risk factor discovery for the frailty syndrome. BMC Med Inform Decis Mak.

[13] Asthana S, Prime S (2023). The role of digital transformation in addressing health inequalities in coastal communities: barriers and enablers. Front Health Serv.

[14] Oksavik JD, Aarseth T, Solbjør M, Kirchhoff R (2021). 'What matters to you?' Normative integration of an intervention to promote participation of older patients with multi-morbidity - a qualitative case study. BMC Health Serv Res.

[15] Tzeng HM, Franks HE, Passy E (2024). Facilitators and barriers to implementing the 4Ms framework of age-friendly health systems: a scoping review. Nurs Rep.

[16] Lesser S, Zakharkin S, Louie C, Escobedo MR, Whyte J, Fulmer T (2022). Clinician knowledge and behaviors related to the 4Ms framework of Age-Friendly Health Systems. J Am Geriatr Soc.

[17] Adler-Milstein JR, Krueger GN, Rosenthal SW, Rogers SE, Lyles CR (2023). Health system approaches and experiences implementing the 4Ms: insights from 3 early adopter health systems. J Am Geriatr Soc.

[18] Straßner C, Frick E, Stotz-Ingenlath G, Buhlinger-Göpfarth N, Szecsenyi J, Krisam J, Schalhorn F, Valentini J, Stolz R, Joos S (2019). Holistic care program for elderly patients to integrate spiritual needs, social activity, and self-care into disease management in primary care (HoPES3): study protocol for a cluster-randomized trial. Trials.

[19] Rathert C, Wyrwich MD, Boren SA (2013). Patient-centered care and outcomes: a systematic review of the literature. Med Care Res Rev.

[20] Kitson A, Marshall A, Bassett K, Zeitz K (2013). What are the core elements of patient-centred care? A narrative review and synthesis of the literature from health policy, medicine and nursing. J Adv Nurs.

[21] Håkansson Eklund J, Holmström IK, Kumlin T, Kaminsky E, Skoglund K, Höglander J, Sundler AJ, Condén E, Summer Meranius M (2019). "Same same or different?" A review of reviews of person-centered and patient-centered care. Patient Educ Couns.

[22] Gutnick D, Hamilton C, Hill N, Howard-Eddings T, Chaya J, McGuire A, Damrow P, Hunter T, Klaber B (2020). Implementation toolkit: what matters to you?. Montefiore Hudson Valley Collaborative.

[23] Berntsen GK, Gammon D, Steinsbekk A, Salamonsen A, Foss N, Ruland C, Fønnebø V (2015). How do we deal with multiple goals for care within an individual patient trajectory? A document content analysis of health service research papers on goals for care. BMJ Open.

[24] Leiter MP, Spence Laschinger HK (2006). Relationships of work and practice environment to professional burnout: testing a causal model. Nurs Res.

[25] Olayiwola JN, Willard-Grace R, Dubé K, Hessler D, Shunk R, Grumbach K, Gottlieb L (2018). Higher perceived clinic capacity to address patients' social needs associated with lower burnout in primary care providers. J Health Care Poor Underserved.

[26] Coulter A, Entwistle VA, Eccles A, Ryan S, Shepperd S, Perera R (2015). Personalised care planning for adults with chronic or long-term health conditions. Cochrane Database Syst Rev.

[27] You EC, Dunt D, Doyle C, Hsueh A (2012). Effects of case management in community aged care on client and carer outcomes: a systematic review of randomized trials and comparative observational studies. BMC Health Serv Res.

[28] Gama F, Tyskbo D, Nygren J, Barlow J, Reed J, Svedberg P (2022). Implementation frameworks for artificial intelligence translation into health care practice: scoping review. J Med Internet Res.

[29] Kirchner JE, Smith JL, Powell BJ, Waltz TJ, Proctor EK (2020). Getting a clinical innovation into practice: an introduction to implementation strategies. Psychiatry Res.

[30] Russell RG, Lovett Novak L, Patel M, Garvey KV, Craig KJ, Jackson GP, Moore D, Miller BM (2023). Competencies for the use of artificial intelligence-based tools by health care professionals. Acad Med.

[31] Mehandru N, Miao BY, Almaraz ER, Sushil M, Butte AJ, Alaa A (2024). Evaluating large language models as agents in the clinic. NPJ Digit Med.

[32] Singhal K, Azizi S, Tu T, Mahdavi SS, Wei J, Chung HW, Scales N, Tanwani A, Cole-Lewis H, Pfohl S, Payne P, Seneviratne M, Gamble P, Kelly C, Babiker A, Schärli N, Chowdhery A, Mansfield P, Demner-Fushman D, Agüera Y Arcas B, Webster D, Corrado GS, Matias Y, Chou K, Gottweis J, Tomasev N, Liu Y, Rajkomar A, Barral J, Semturs C, Karthikesalingam A, Natarajan V (2023). Large language models encode clinical knowledge. Nature.

[33] Olsen CF, Bergland A, Debesay J, Bye A, Langaas AG (2021). Patient flow or the patient's journey? Exploring health care providers' experiences and understandings of implementing a care pathway to improve the quality of transitional care for older people. Qual Health Res.

[34] Rubenstein LZ, Josephson KR, Wieland GD, English PA, Sayre JA, Kane RL (1984). Effectiveness of a geriatric evaluation unit — a randomized clinical trial. N Engl J Med.

[35] Kumar GS, Klein R (2013). Effectiveness of case management strategies in reducing emergency department visits in frequent user patient populations: a systematic review. J Emerg Med.

[36] de Bruin SR, Versnel N, Lemmens LC, Molema CC, Schellevis FG, Nijpels G, Baan CA (2012). Comprehensive care programs for patients with multiple chronic conditions: a systematic literature review. Health Policy.

[37] Low LF, Yap M, Brodaty H (2011). A systematic review of different models of home and community care services for older persons. BMC Health Serv Res.

[38] Huntley AL, Thomas R, Mann M, Huws D, Elwyn G, Paranjothy S, Purdy S (2013). Is case management effective in reducing the risk of unplanned hospital admissions for older people? A systematic review and meta-analysis. Fam Pract.

[39] Wang D, Liu P, Zheng Y, Qiu X, Huang X (2025). Heterogeneous graph neural networks for extractive document summarization. ArXiv. Preprint posted online on April 26, 2020.

[40] Papalampidi P, Lapata M (2025). Hierarchical3D adapters for long video-to-text summarization. ArXiv. Preprint posted online on October 10, 2022.

[41] Finkler SA, Knickman JR, Hendrickson G, Lipkin M Jr, Thompson WG (1993). A comparison of work-sampling and time-and-motion techniques for studies in health services research. Health Serv Res.

[42] Lopetegui M, Yen PY, Lai A, Jeffries J, Embi P, Payne P (2014). Time motion studies in healthcare: what are we talking about?. J Biomed Inform.

[43] Kraišniković C, Harb R, Plass M, Zoughbi WA, Holzinger A, Müller H (2025). Fine-tuning language model embeddings to reveal domain knowledge: an explainable artificial intelligence perspective on medical decision making. Eng Appl Artif Intell.

[44] Oosthuizen D (2022). Investigating the usability and quality of experience of mobile video-conferencing apps among bandwidth-constrained users in South Africa. University of Cape Town.

[45] Brooke J (1996). SUS: a 'quick and dirty' usability scale. Usability Evaluation In Industry.

[46] Keogh A, Dorn JF, Walsh L, Calvo F, Caulfield B (2020). Comparing the usability and acceptability of wearable sensors among older Irish adults in a real-world context: observational study. JMIR Mhealth Uhealth.

[47] Bikker AP, Fitzpatrick B, Murphy D, Mercer SW (2015). Measuring empathic, person-centred communication in primary care nurses: validity and reliability of the Consultation and Relational Empathy (CARE) Measure. BMC Fam Pract.

[48] Balestroni G, Bertolotti G (2012). [EuroQol-5D (EQ-5D): an instrument for measuring quality of life]. Monaldi Arch Chest Dis.

[49] Elwyn G, Barr PJ, Grande SW, Thompson R, Walsh T, Ozanne EM (2013). Developing CollaboRATE: a fast and frugal patient-reported measure of shared decision making in clinical encounters. Patient Educ Counsel.

[50] Steger MF, Frazier P, Oishi S, Kaler M (2006). The meaning in life questionnaire: assessing the presence of and search for meaning in life. J Counsel Psychol.

[51] Schulenberg SE, Strack KM, Buchanan EM (2011). The Meaning In Life Questionnaire: psychometric properties with individuals with serious mental illness in an inpatient setting. J Clin Psychol.

[52] Abacha AB, Yim WW, Adams G, Snider N, Yetisgen-Yildiz M (2023). Overview of the MEDIQA-chat 2023 shared tasks on the summarization and generation of doctor-patient conversations. Proceedings of the 5th Clinical Natural Language Processing Workshop.

[53] Ando K, Okumura T, Komachi M, Horiguchi H, Matsumoto Y (2022). Is artificial intelligence capable of generating hospital discharge summaries from inpatient records?. PLOS Digit Health.

[54] Kvael LA, Debesay J, Bye A, Bergland A (2019). Health-care professionals' experiences of patient participation among older patients in intermediate care-at the intersection between profession, market and bureaucracy. Health Expect.

[55] Lo B, Almilaji K, Jankowicz D, Sequeira L, Strudwick G, Tajirian T (2021). Application of the i-PARIHS framework in the implementation of speech recognition technology as a way of addressing documentation burden within a mental health context. AMIA Annu Symp Proc.

[56] Hodgson T, Magrabi F, Coiera E (2017). Efficiency and safety of speech recognition for documentation in the electronic health record. J Am Med Inform Assoc.

[57] Sharma M, Tong M, Korbak T, Duvenaud D, Askell A, Bowman SR, Cheng N, Durmus E, Hatfield-Dodds Z, Johnston SR, Kravec S, Maxwell T, McCandlish S, Ndousse K, Rausch O, Schiefer N, Yan D, Zhang M, Perez E (2025). Towards understanding sycophancy in language models. ArXiv. Preprint posted online on October 20, 2023.

[58] Nashwan AJ, Abujaber AA, Choudry H (2023). Embracing the future of physician-patient communication: GPT-4 in gastroenterology. Gastroenterol Endosc.

[59] Kocaballi A, Ijaz K, Laranjo L, Quiroz JC, Rezazadegan D, Tong HL, Willcock S, Berkovsky S, Coiera E (2020). Envisioning an artificial intelligence documentation assistant for future primary care consultations: a co-design study with general practitioners. J Am Med Inform Assoc.

[60] (2024). What matter's to you? Building relationships to improve the patient experience metrics and employee engagement. The Beryl Institute.

[61] A guide to having conversations about what matters. BC Patient Safety & Quality Council.

[62] Holgersson M, Björkdahl J, Essén A, Frishammar J (2024). Health care platforms need a strategy overhaul. MIT Sloan Management Review.

[63] Cicirelli G, Marani R, Petitti A, Milella A, D'Orazio T (2021). Ambient assisted living: a review of technologies, methodologies and future perspectives for healthy aging of population. Sensors (Basel).

[64] Meredith SJ, Roberts H, Grocott MP, Jack S, Murphy J, Varkonyi-Sepp J, Bates A, Lim SE (2023). Frail2Fit study protocol: a feasibility and acceptability study of a virtual multimodal intervention delivered by volunteers to improve functional outcomes in older adults with frailty after discharge from hospital. BMJ Open.

[65] Kime N, Wright A, Heaven A, Hawkins R, Smith J, Cundill B, Foy R, Lawton R, Farrin A, Hulme C, Clegg A (2022). Implementing personalised care planning for older people with frailty: a process evaluation of the PROSPER feasibility trial. BMC Geriatr.

[66] Sait MI, Christie RA, Cox C, Board M, Thomas S, O'Sullivan C, Davies C, Walker DM, Vassallo M, Sadler EA, Allen-Pick M, Moore P, Bradbury K, Murphy J (2024). DIALOR (DIgitAL cOaching for fRailty): protocol for a single-arm mixed-methods feasibility study of a digital health coaching intervention for older people with frailty in primary care. BMJ Open.

